# Design Strategies for Virtual Reality Interventions for Managing Pain and Anxiety in Children and Adolescents: Scoping Review

**DOI:** 10.2196/14565

**Published:** 2020-01-31

**Authors:** Naseem Ahmadpour, Melanie Keep, Anna Janssen, Anika Saiyara Rouf, Michael Marthick

**Affiliations:** 1 Design Lab Sydney School of Architecture, Design and Planning The University of Sydney Sydney Australia; 2 Faculty of Health Sciences The University of Sydney Sydney Australia; 3 Research in Implementation Science and eHealth Group Charles Perkins Centre The University of Sydney Sydney Australia; 4 School of Life and Environmental Sciences Charles Perkins Centre The University of Sydney Sydney Australia

**Keywords:** virtual reality, distraction, pain, anxiety, children, adolescents, design

## Abstract

**Background:**

Virtual reality (VR) technology has been explored in the health sector as a novel tool for supporting treatment side effects, including managing pain and anxiety. VR has recently become more available with the launch of low-cost devices and apps.

**Objective:**

This study aimed to provide an updated review of the research into VR use for pain and anxiety in pediatric patients undergoing medical procedures. Specifically, we wanted to gain an understanding of the techniques and goals used in selecting or designing VR apps in this context.

**Methods:**

We performed a scoping review. To identify relevant studies, we searched three electronic databases. Two authors screened the titles and abstracts for relevance and eligibility criteria.

**Results:**

Overall, 1386 articles published between 2013 and 2018 were identified. In total 18 articles were included in the review, with 7 reporting significant reduction in pediatric pain or anxiety, 3 testing but finding no significant impact of the VR apps employed, and the rest not conducting any test of significance. We identified 9 articles that were based on VR apps specifically designed and tailored for pediatric patients. The findings were analyzed to develop a holistic model and describe the product, experience, and intervention aspects that need to be considered in designing such medical VR apps.

**Conclusions:**

VR has been demonstrated to be a viable choice for managing pain and anxiety in a range of medical treatments. However, commercial products lack diversity and meaningful design strategies are limited beyond distraction techniques. We propose future VR interventions to explore skill-building goals in apps characterized by dynamic feedback to the patient and experiential and product qualities that enable them to be an active participant in managing their own care. To achieve this, design must be part of the development.

## Introduction

### Background

In recent years, virtual reality (VR) technology has been explored in the health sector as a novel tool for supporting and monitoring treatment [1]. This increased interest in VR reflects recent advancements in commercial VR headsets (eg, Oculus Rift by Oculus VR, LLC) and mobile VR capabilities (eg, Samsung Gear VR by Samsung Electronics Co), leading to more affordable and feasible implementation of VR in health care. This use of VR in health care was pioneered by Hoffman et al, who, in the early 2000s, created SnowWorld, a VR gaming system that was able to reduce pain perception during burn wound care in both adolescent [2] and adult patients [3]. Since then, a number of studies have demonstrated the effectiveness of VR as a nonpharmacologic intervention for managing treatment side effects, such as pain, anxiety, or distress [4]. Typically, VR interventions involve a head-mounted display (HMD) worn by users, which allows them to experience 3D content (eg, videos and games) in an immersive virtual environment (VE). VR technology can afford health care providers greater capacity to improve patients’ adherence to painful or distressing procedures while reducing the risk of the side effects associated with pharmacologic alternatives such as opioid dependency in pain management [5]. As a consequence, VR technology can improve patients’ quality of life and satisfaction with care [6]. A recent systematic review and meta-analysis of 20 studies found evidence in support of significant reduction of acute pain in adults, with promising results for chronic pain [7]. Another review found similar evidence of efficacy for VR as an analgesic; however, it revealed a need for more research into mechanisms underpinning the success of VR apps to inform the design of future VR systems [8].

Pain and anxiety management using VR is often hypothesized to occur through distraction, by diverting a person’s attention away from painful stimuli [9]. According to the neuromatrix theory of pain [10], cognitive, sensory, and affective inputs as well as factors influencing those, such as attention, can change pain perception and ultimately a person’s response to it. Accordingly, by engaging the cognitive resources of a person in a task (eg, by watching or playing something through VR), offering them sensory stimulation (eg, visual and auditory), or offering them positive affective experiences (eg, enjoyment or success), limited capacity remains for the person to process or attend to pain [11].

Alternative applications of VR for managing pain and anxiety are explored through nondistraction techniques and tested in a range of settings from experimental to treatment (eg, fibromyalgia, burn wound care, and pediatric chronic headache) [12]. Instead of diverting a person’s attention to unrelated tasks, those applications suggest that VR is effective when used to empower the user by allowing them to directly control their affective response to the painful stimuli. For instance, Loreto-Quijada et al [13] designed a VR app that enabled the user to manipulate unpleasant audio-visual aspects of a virtual object to create a more pleasant form and achieve a sense of calm and relief in the process. In another example, Shiri et al [14] created a virtual doppelgangers app to support the treatment of pediatric chronic headache. This involved a photorealistic capture of a user’s facial expression combined with biofeedback to mimic their real-time emotional state. The app mediated an exercise of relaxation by bringing the user’s attention to self and requiring them to focus on changing the virtual facial expression from a state of agony to calm [14].

The recent strategies for VR interventions in pain and anxiety management have yet to be systematically compared. This is needed to design VR experiences that are effective for use in health care and offer patients new opportunities for self-reflection and lasting impact on pain management capabilities. Beyond distraction therapy, there is currently little variation among VR strategies for pain and anxiety treatment [12]. VR technology is increasingly accessible to consumers who will be less impressed by the novelty of this technology and its power to *distract* in the near future. We will soon need better design strategies and the ability to predict the outcomes of VR intervention to help individuals with complicated health needs. Currently, though, there is limited understanding of the key design elements of effective VR experiences for health care. A model for understanding the strategies underlying VR for pain and anxiety management is needed to guide the design of successful VR interventions for patients across ages, health conditions, interaction constraints, and well-being goals. This study presents findings from a scoping review on VR for managing pain and anxiety in pediatric patients and synthesizes them to propose a model for designing such apps.

### Objectives

This study followed a scoping review methodology specified by Arksey and O’Malley [15]. The scoping review aimed to investigate the breadth of the design and application of VR technology used for managing pain and anxiety in pediatric patients undergoing medical procedures. The outcome is summarized to identify a gap in the existing literature. We have chosen this population, as there is currently little evidence to support the systematic design of tailored VR apps. Recent research has indicated the increasing awareness of children about VR technology, as a survey of 1917 children aged 2 to 15 years in the United States found that only 18.9% (364/1917) of them had not heard of VR technology, and 43.9% (843/1917) of participants expressed extreme interest in VR [16]. Therefore, it is important to understand how we can leverage a technology well known to children for its potential health benefits.

The search was limited to articles published after 2012, the year the commercial Oculus Rift VR technology was first announced, as the launch of this device made VR technology readily accessible and available. The scoping review explored three questions: (1) What are the proposed intervention techniques and goals for VR apps to reduce pain and anxiety in pediatric populations? (2) What are the design considerations for VR experiences for reducing pain and anxiety around various medical procedures in pediatric populations? and (3) What tools and evaluation methods were used for assessing the efficacy of VR?

These questions are developed to inform the design of future VR apps for pediatric patients to reduce pain and anxiety. Recent literature suggested that although there is sufficient evidence on the efficacy of VR for reducing pain and anxiety, there is a visible gap in research on guiding strategies for designing such VR apps [8]. Through our first two research questions (RQs), we aim to understand the details on design directions and guiding principles of VR as an analgesic for pediatric population. The third RQ investigates how the designed apps are evaluated to reveal potential gaps in the assessment of the intervention and the outcomes. Findings from the scoping review were then synthesized and used for developing a model on the design elements that characterize VR apps for managing pain and anxiety.

## Methods

### Search Strategy and Eligibility Criteria

A literature search was undertaken between October 2018 and December 2018 in Scopus, Association for Computing Machinery (ACM), and PsycINFO, using combinations and variations of the following search terms: children, adolescent, virtual reality, distraction, and anxiety, stress, and pain. A detailed search strategy is provided in Multimedia Appendix 1. The search terms were chosen to align with the research aims and retrieve articles that use VR technology (bespoke design and commercial) in a wide range of contexts. The databases were chosen as those were more likely to include publications relevant to the RQs, given the exploratory nature of this research into the principles that informed the design of the VR apps. In particular, the ACM digital library includes publications from the fields of interaction design and human-computer interaction that are likely to explore bespoke VR designs. The inclusion of studies was then carried out as follows.

Peer-reviewed articles, published in English after 2012, and describing a VR intervention for managing or reducing, or both, pain, anxiety, or stress, or their combination, in children and adolescents (aged <18 years) undergoing medical procedures or treatment were included. Studies that investigated the use of VR for patient education, social skills training, or rehabilitation were not included. Studies that specifically addressed interventions for managing phobias were also excluded, as the underlying treatment is often long term and vastly different to the management of pain or anxiety. Papers were excluded if they focused on infants or children with intellectual disability, autism spectrum disorder, cerebral palsy, neurodevelopmental disorders or permanent cognitive disorders, or physical impairment. Those studies were excluded because the interventions would potentially involve strategies specific to a condition or a population group and may not be possible to generalize.

### Study Selection

**Figure 1:**
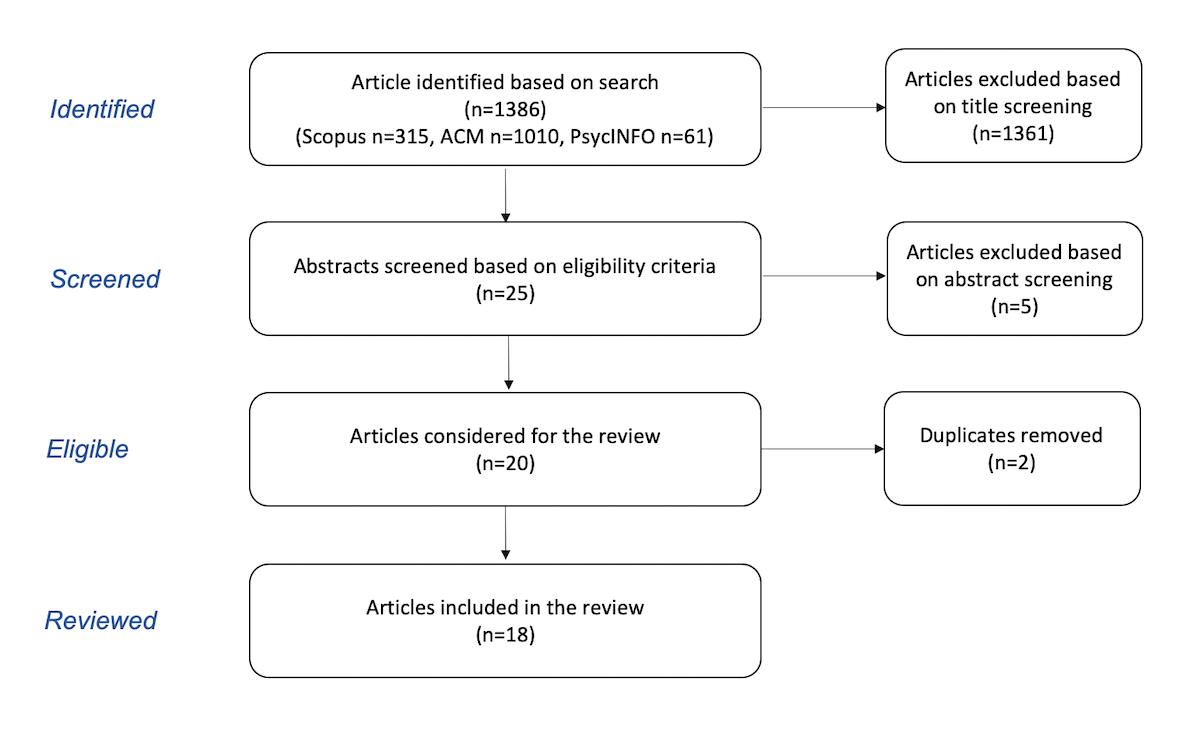
Flowchart of the literature screening and selection process. ACM: Association for Computing Machinery.

Overall, 1386 articles were identified. Two authors screened the titles and abstracts for relevance and eligibility criteria. If a decision could not be made based on abstract text, the full text was retrieved to make the assessment. After the title and abstract review, 20 articles were selected for a full-text review, of which 2 were duplicates and were removed. In total, 18 articles were included in the final shortlist for the scoping review. The elimination process is shown in Figure 1.

### Evaluation Process

A data collection form was developed to extract the following study details: authors, year, medical procedure being addressed, sample size, age range, gender distribution, type of headset used, commercial product or bespoke design, description of VR app, description and design qualities of VR app (if available), study design, control group (if present), intervention goal, dependent variable, tools for measurement, and a summary of results. Those details allowed us to generate information relevant to the three RQs and specifically identify the intervention techniques and goals relevant to each VR app (RQ1), the design considerations for the app and the technology (RQ2), and trials and assessment details (RQ3). The key characteristics of the included studies are summarized qualitatively and tabulated in Multimedia Appendix 2.

## Results

### Scoping Review Results

Among the 18 articles that were reviewed, VR interventions for pain or anxiety were attributed to a wide range of medical treatment in children: magnetic resonance imaging (MRI) and radiology examinations [17]; wound debridement [18], specifically, burn wound [19-22]; venipuncture [23,24]; phlebotomy or blood draw [25,26]; cold pressor experiment [27]; preoperative care in hospital and anesthesia [28,29]; cancer treatment and chemotherapy [30,31]; injection, for instance, botulinum toxin [32]; and local anesthesia in dental care [33]. One study described the design of a VR app for broadly defined medical procedures, without specifying one [34].

Among those that specified a procedure, 7 articles addressed pain directly [18,21,24,26,33], whereas 6 articles addressed anxiety associated with pain during a procedure [20,22,23,25,32]. In addition, 4 articles addressed anxiety linked to a medical procedure [17,28-30].

The average age range specified for children and adolescents in the selected articles varied between 5 and 21 years, and one study reported participants aged between 8 and 57 years [18]. In terms of equipment, 10 articles used mobile phones to deploy VR apps on low-cost headsets, such as Google Cardboard, Samsung Gear, and View Master. One article described the design of a 360° video tour of the operation room and suggested that the outcome can be used on any platform [28]. Six articles used commercial high-end VR HMD, such as Oculus Rift and Sony HMZ T2. One article involved adolescents with burn injuries to head and, therefore, used a Kaiser Optics SR80a VR helmet mounted on a custom-built, articulated-arm tripod device to avoid any further wound damage because of wearing HMD. The details relevant to specific research questions are discussed in the following sections.

#### Research Question 1: What Are the Proposed Intervention Techniques and Goals for Virtual Reality Apps to Reduce Pain and Anxiety?

We reviewed the articles to identify any intervention goal (and relevant techniques to achieve those goals) that were specified or somewhat described and were used to select or design the VR technology. In total, 9 studies employed commercially available content, among which 4 studies used *SnowWorld*. As this game has been widely used in the past two decades, it is categorized as a commercial VR app. A total of 9 articles discussed a bespoke VR app specifically tailored for children and adolescent patient populations. We then classified the goals into three categories: those that aimed to distract the patient, those that aimed to strategically shift the patient’s attention, and those that aimed to help patients build capacities to modulate pain through the use of the VR app. The studies in each category are discussed in the following paragraphs.

Several studies cited distraction as the main goal of the intervention; that is, attentional resources are temporarily engaged through VR, so there is limited capacity remaining for processing pain or anxiety-inducing stimulus. In its simplest form, distraction therapy involved entertaining media (eg, watching videos) [21,26,32,33]. Others used interactive games, such as SnowWorld game [18,24], Nintendo Wii *Sonic and the Secret Rings* game [27], and *Bear Blast* developed by AppliedVR [25]. The degree of interaction varied among those games; SnowWorld and Sonic and the Secret Rings games are played using a hand controller, whereas Bear Blast involves hands-free and gaze-based interaction (users gaze at a direction to move or perform in-game tasks).

A number of games were characterized as distraction therapy but cited more specific focus-shifting techniques for modulating pain or anxiety output. For instance, Birnie et al [30] suggested that enabling the user to maintain a point of focus on an object in front of them is important and designed a game where children can shoot rainbow balls at moving objects. Furthermore, Piskorz and Czub [23] used a Multiple Object Tracking paradigm to set priorities for cognition and shifting the player’s attention from one virtual object to another during a venipuncture procedure. Players engaged with that task through head movement and gaze-based interaction.

Grishchenko et al [34] suggested a strategy to build further capacity in patients by creating a fixed reference point in the frame of view while facilitating deep breathing through game mechanics. Their gaze-based game, *Voxel Bay*, used audio command to make the game interactive and help players exhale more deeply to help prevent hyperventilation, a common side effect in children receiving painful treatment. Grishchenko et al [34] also suggested that relaxing audio can assist with regulating pain or anxiety and block anxiety-inducing noises of medical treatment. This point was also emphasized by Birnie et al [30] and Ko et al [22]. Finally, some articles used exposure to potentially distressing stimulus in a safe environment to alleviate fear and anxiety before the medical appointment. Liszio and Masuch [17] designed a game to introduce children to their MRI appointment. O’Sullivan et al [28] and Ryu et al [29] created 360° video tours of the hospital to familiarize children with anesthesia procedure before their operation.

#### Research Question 2: What Are the Design Considerations for Virtual Reality Experiences for Reducing Pain and Anxiety Around Various Medical Procedures in Pediatric Populations?

We identified three clear groups of considerations that guided the design or selection of VR apps in review articles: creating a perception of autonomy and control for the user, providing a perception of safety by using familiar elements, and fostering empathy through the narrative. Further considerations linked to those groups also emerged, as discussed below.

An example of the first design consideration, autonomy and control, is the VR app developed by Birnie et al [30] to reduce needle-related anxiety in young cancer patients. The product was created through an iterative participatory design process with direct input from children, resulting in a game of treasure hunt and shooting rainbow balls at underwater creatures. Birnie et al [30] described principles, such as simplicity, interactivity, and aesthetic qualities such as colorful environment, which are important for mediating user engagement. Those principles seem to be relevant to the design of the product (the VR app). The researchers also suggested considerations important for creating a good experience for the user. For instance, they found that the ability to control the exploration within the game environment and to have fun impacts the user’s sense of *presence* in the VE, which is crucial for increased attention to distractors and, therefore, the efficacy of the VR intervention [30]. Similarly, Ng et al [31] suggested that it is important that VR apps help cancer patients overcome boredom to manage treatment side effects. In another example, Gold and Mahrer [25] suggested that product aspects such as the complexity of a player’s in-game progress can keep children and adolescents engaged during procedural distraction by eliminating worry about their performance. Gold and Mahrer [25] used Bear Blast, a commercial game where the user controls a firing cannon in VE and enters a new level every 2.5 min. Similar to the VR app developed by Birnie et al [30], Bear Blast intends to facilitate experience aspects such as positive reinforcement of *experimentation and activity* [25]. The importance of experiencing a sense of empowerment and autonomy was also emphasized by Grishchenko et al [34] in their game Voxel Bay, an audio command–activated game where deep exhales (into a microphone) assist the player to move a boat in VE. To keep the player interested and, therefore, distracted, Voxel Bay employs a narrative whereby the player controls some elements of their adventure (through playing diverse short games) but also experiences surprise, as the clinician controls the overall game navigation from their remote control station [34].

The second design consideration, using familiar elements to elicit a sense of safety, was demonstrated in familiar product qualities or creating familiarity through the experience. Familiar product qualities were often linked to featured elements in VE, such as references to popular games (eg, Minecraft and Lego [34] or SimCity [31]). Creating familiarity through the experience then involved creating a precedence for the medical procedure, for instance, by locating the game in the hospital environment [17,28,29]. This type of familiarization aimed to eliminate cognitive appraisal of elements that may be perceived negatively by children and adolescents and allow them to safely prepare for their appointment at a convenient time and place.

The third design consideration pertained to creating comfort for the patient by fostering a sense of empathy in VE. A number of games employed in-game companion characters to achieve that. For instance, Grishchenko et al [34] allowed children to choose an animal character to accompany them on a journey, and Ng et al [31] designed a companion cow character in a virtual farm for cancer patients to care for during chemotherapy. Ng et al [30] argued that symbolizing hope and growth and providing everyday motivation within the game (to interact and collect rewards) can enhance a sense of well-being in players with life-altering conditions such as cancer and used metaphors such as growing vegetables to signify that. Overall, narrative elements were used in all bespoke apps that were purposefully designed for children and adolescents. Some examples included hunting for treasures along a journey [27,34]; adventure [22,30]; playful interaction with animal characters; farming vegetables [31]; and shooting snowballs [18,24] or rainbow balls [30], or cannons [25] at animated objects.

#### Research Question 3: What Tools and Evaluation Methods Were Used for Assessing the Efficacy of Virtual Reality Interventions for Pain and Anxiety in Children?

In all, 10 articles used some form of control group in testing (eg, baseline control); 7 of which were randomized controlled trials, with sample sizes ranging between 13 and 143 participants. Without the assessment of usability or user experience, there is little evidence on the impact of design choices on patient experience or ability to manage pain. Outcomes were not consistently or systematically assessed among the reviewed studies. In all, 3 papers described the design of a VR app but did not test the outcome [22,28,34]. Others used interview and focus groups [31] and feedback on the usability and experiences of users [21] or caregivers [32] and used that feedback to tailor the design to children’s needs [30]. One article presented a case study with one participant [19].

Overall, 7 studies showed that VR interventions significantly reduced anxiety [29], pain [18,20,24,27], or both [23,25]; and 3 studies tested but found no significant impact of the VR intervention [17,26,33].

All studies that tested anxiety and pain outcomes used self-report, mostly, standardized anxiety (eg, State-Trait Anxiety Inventory for Children and Yale Preoperative Anxiety Scale), pain severity (eg, through Visual Analogue Thermometer and Adolescent Pediatric Pain Tool), and worry related to pain (Wong-Baker Faces Pain Scale) scales. In some cases, numeric scales were developed by researchers to measure pain, nausea, stress, or satisfaction. A number of studies relied on expert evaluation of observed pain indicators by using tools such as Faces, Legs, Activity, Cry, and Consolability [32]. One study [24] evaluated the impact of VR intervention on multiple aspects of pain: cognitive component (time spent thinking about pain), affective component (unpleasantness), and perceptive component (worst pain). Only one study combined self-report with physiological assessment of pain output through pulse rate [33].

### Summary of Findings

A range of considerations were identified through our literature review that are important for creating bespoke VR apps for pain and anxiety management. These are summarized in Table 1 and include two categories of considerations for creating virtual reality apps: intervention considerations (underlined by three types of goals, namely distraction, focus shifting, and skill building) and design considerations. A close examination of the findings relevant to RQ1 and RQ2 reveals that some design considerations are specific to the product aspects (the VR app and the technology and its content), whereas other design considerations are specific to the aspects of experience that the patient might have as the result of interacting with the product (such as their emotional experience and enjoyment). Examples and articles relevant to each of those categories are provided.

**Table 1:** Classification of findings relevant to considerations for creating virtual reality apps for managing pain and anxiety.

Considerations		Examples	Articles
**Intervention considerations**			
	Distraction	Engaging the patient’s attention through VR^a^ content through entertaining videos or games, for example, *Sonic and the Secret Rings* (Nintendo Wii) and *SnowWorld*	Faber et al [18], Hoffman et al [19], Jeffs et al [20], Scapin et al [21], Atzori et al [24], Gold and Mahrer [25], Gerçeker et al [26], Chau et al [32], Al-Habibi et al [33]
	Focus shifting	Engaging the patient’s cognitive resources through game-led tasks, for example, multiple object tracking	Piskorz and Czub [23], Birnie et al [30]
	Capacity or skill building	Engaging the patient in game-led activities to build capacities for self-regulation, for example, deep breathing in *Voxel Bay*	Liszio and Masuch [17], O’Sullivan et al [28], Ryu et al [29], Grishchenko et al [34]
**Design considerations**			
	Product	Tailoring simplicity and interactivity to improve control Removing barriers to engagement to improve a sense of autonomy Diversifying sensory inputs Diversifying sensory output and stimulation Improving sense of presence	Gold and Mahrer [25], Birnie et al [30], Ng et al [31], Grishchenko [34]
	Experience	Improving ability to attend to distractors Creating a sense of safety through familiar design elements or familiarity with medical procedure Incorporating narrative elements Cultivating growth and motivation Fostering empathy and compassion	Liszio and Masuch [17], Jeffs et al [20], Ko et al [22], Atzori et al [24], Gold and Mahrer [25], Sil et al [27], O’Sullivan et al [28], Ryu et al [29], Birnie et al [30], Ng et al [31], Grishchenko et al [34]

^a^VR: virtual reality.

Product aspects for designing VR apps for managing pain or anxiety in children and adolescents can be achieved in several ways, for instance, by (1) tailoring simplicity and interactivity of VR for young users, taking into account aesthetic qualities that conform to the user’s expectations and prior experiences with digital technologies (eg, colorful environment inspired from familiar themes); (2) removing barriers to engagement to allow young players to proceed in game regardless of their performance; (3) diversifying sensory input modalities in user-device interaction, for instance, by combining audio commands with gaze input to instigate a game action; (4) diversifying sensory stimulation, for instance, by incorporating music, to further divert attention from painful stimuli; and (5) improving immersion in VE by combining sensory (audio, visual, and tactile) and affective (eg, fun) experiences in active interaction with virtual objects in a game environment to help users be immersed in VR.

The experience aspects can be tailored in multiple ways, for instance, by (1) empowering users with the ability to attend to distractors through engaging features; (2) eliciting comfort and positive affect through interpersonal interaction (eg, doctors control some game elements) or familiarity (eg, familiar design elements and understanding medical procedure)—our review suggests that a full spectrum of human affect (eg, positive emotions such as pride) is not explored in many examples we reviewed, and there is scope for further investigation; (3) incorporating narrative elements (eg, treasure hunt); (4) enhancing motivation through cultivating personal growth (eg, introducing daily incentives such as caring for a garden); and (5) fostering empathy and companionship, for instance, through virtual companion characters in games.

These aspects, in combination with the intervention aspects (such as goals), can be used to characterize a VR intervention for mediating self-management of pain or anxiety. The intervention aspects include strategies informed by the three identified goals for managing pain and anxiety. Those may involve a varying degree of user engagement from passive participation (eg, watching a video, with no interaction and receiving no feedback) to active (eg, interactive games, providing interactive feedback to the user, which can align with a pain management strategy). We distinguish between distraction and focus shifting in VR, as the latter involves active participation in cognitive tasks with feedback to guide the user’s interaction and consequently their response to pain. Typical examples of focus shifting in VR offer one or multiple *points of focus* for users (eg, multiple object tracking) within a game environment, for example, treasure hunt. Some VR interventions go even further, engaging the patient in active self-regulation, for example, relaxation through deep breathing in Voxel Bay [34]. Evidence on those types of intervention is scarce (*Voxel Bay* was not tested in a clinical trial), and more research is needed to examine the range and effectiveness of such interventions.

**Figure 2:**
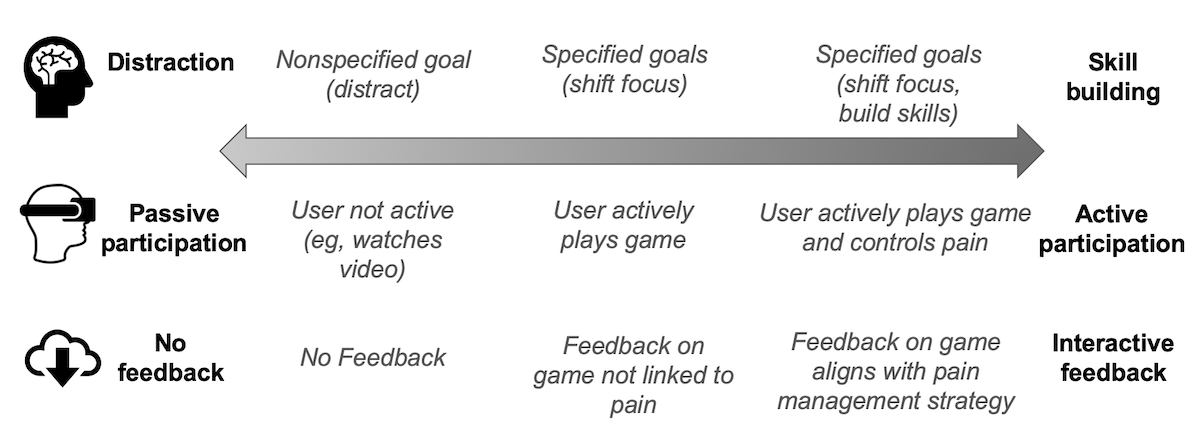
VR interventions for pain and anxiety on a continuum; characterized in terms of patient experience and role (passive to active), VR design (feedback to user from none to interactive feedback) and intervention goal (distraction to skill-building).

In an attempt to detail the relationship among the components of the holistic model, we propose a continuum to summarize the multitude of intervention strategies for managing pain and anxiety in VR, ranging from distraction to skill building, as shown in Figure 2. This elaborates further on patient experience and role (ranging from passive to active), depending on the intervention goal (ranging from distraction to skill building) and the VR product design (level of feedback provided to the patient).

## Discussion

### Overview

Through reviewing a selection of 18 articles that employed VR technology, we found a number of areas where this technology is currently administered to modulate pain and anxiety in children: acute (eg, wound care) and chronic (eg, cancer treatment) pain or anxiety, needle phobia (eg, blood draw), and procedural care (eg, MRI and preoperative care) in children and adolescents. We searched for goals and considerations to generate insight to support the design of future VR apps for pediatric patients. This resulted in two main contributions. The first is a classification of the range of intervention goals in pain management into distraction, focus shifting, and skill building. This classification is then used to inform our second contribution, a holistic model of considerations for designing medical VR apps.

### A Holistic Model to Support the Design of Medical Virtual Reality Apps

**Figure 3:**
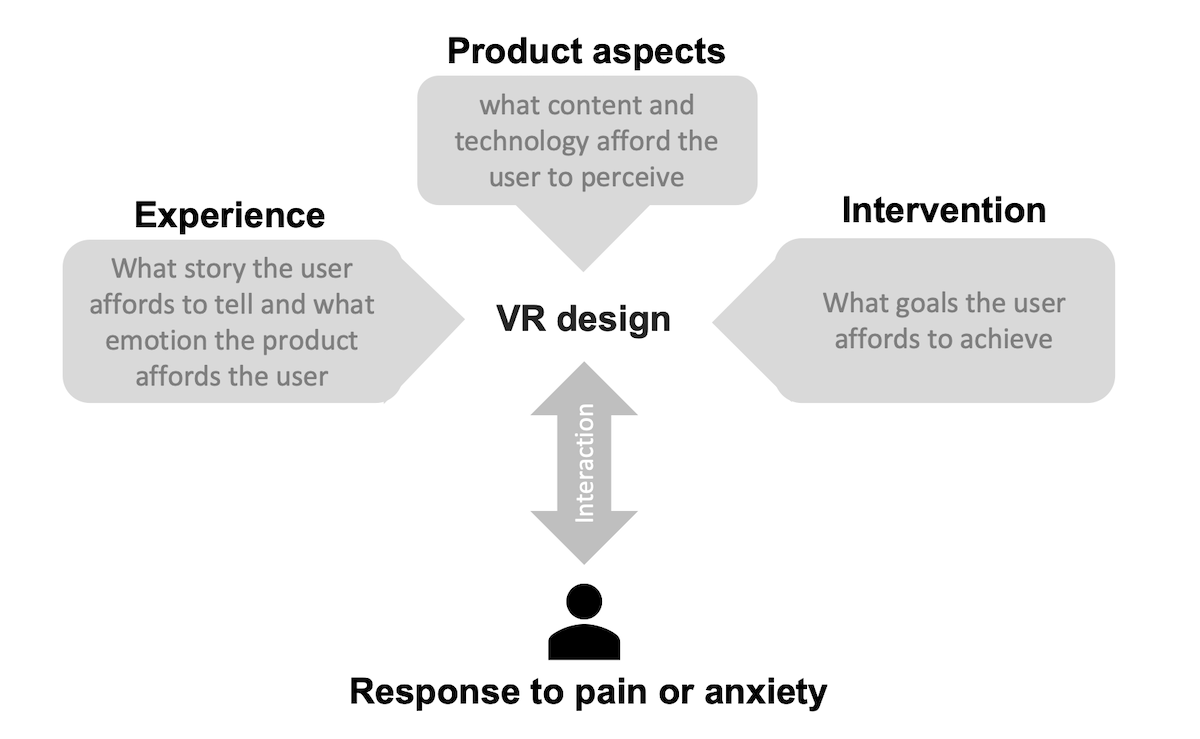
A holistic model to support design of VR interventions for managing pain and anxiety.

The holistic model (shown in Figure 3) proposes that the way a VR product is perceived by the users (product aspects), combined with the experience and emotions it elicits (experience aspects), mediates intervention goals for managing pain or anxiety (intervention aspects). The model is unique, as it aligns a human-centered approach consisting of VR product and experience aspects with a medical approach consisting of intervention aspects appropriate for reducing pain or anxiety. Previous research has discussed elements of VR analgesia beyond distraction [12,13]; however, those fall short of exploring the design opportunities provided by our holistic model. The details and theoretical relevance of each aspect of the model are described in the following paragraphs.

Experience aspects subscribe to the definition of an experience, a multifaceted personal narrative, with a beginning and an end [35], that can be articulated retrospectively and characterized based on interactions, feelings, meanings, and actions [36]. In human-computer interaction research, experiential aspects are attributed to affect and human psychological needs. There are several approaches to defining psychological needs. At a basic level, Peters et al [37] propose fulfilling three needs suggested by the self-determination theory (autonomy, competence, and relatedness) as proxies for a well-being–supportive design. In a study on VR exercise platforms, Ijaz et al [38] found that two of those needs (autonomy and competence) strongly correlate with positive affect (eg, enjoyment) in immersive VR platforms for health and exercise. In technology-mediated experiences, affect (encompassing various constructs with feelings, such as emotions and moods) is often regarded as an integral part of an experience, and positive affect is central to enabling achievement of personal goals for well-being [36]. On the basis of the above, the experience aspects of VR for managing pain and anxiety can be described in terms of the narrative of the experience and emotions that are elicited through interaction.

Product aspects are described in terms of the user’s subjective perceptions of the product content and technology. Hassenzahl et al [36] defined product aspects as how people perceive a product or translate its features (eg, screen and controls), material, style, and interaction form into qualities such as fun or complicated. Hassenzahl [39,40] suggested the way we perceive a product complements our experience of it and identified two main groups of product qualities that contribute to its experience. One group is pragmatic or instrumental qualities (eg, predictable, manageable, and simple) and refers to the judgment of a product’s capability to support what we can do with it. The other group is hedonic qualities (eg, stylish and professional) and refers to how the product supports pleasure and ownership in use [36]. In this paper, we uncovered VR product qualities that are relevant to pain or anxiety management in the literature. For instance, immersion is an important aspect of VR products and defined as sensory stimulations linked to the perception of presence in VE, that is, feeling as if one is there. This was deemed important by some papers [30], as achieving a sensation of presence in VR can override our perception of pain or distressing stimuli and therefore results in better distraction during medical procedures [41]. Hedonic qualities include aesthetic, playful, and adventurous [22,30] or familiar [31,34].

Intervention aspects are linked to the intended health goals and well-being outcomes for the user. Although the ultimate goal for VR interventions in this study is pain or anxiety management, the strategy for achieving such goal may vary from simple distraction to active participation in a self-regulatory process. Liszio and Masuch [17] suggested that an intervention for pain and anxiety management might take a cognitive approach (ie, address the appraisal of the stimulus that is perceived harmful or stressful) or an affective approach (ie, provide an emotional coping strategy for the individual). Therefore, understanding those strategies through specific goals and subgoals provides a roadmap for the intervention and can guide practitioners and researchers in advising treatment regimen and configuring complex VR systems that support self-regulation.

Our proposed holistic model can be used as a lens to examine the trends and evidence of VR interventions. It can also be employed as a proxy to devise design strategies to achieve the intervention goals. There is currently no other model that puts those elements in perspective. As this model is based on evidence from our scoping review, it can be considered feasible for design and interdisciplinary examination of evidence in the fields of interaction design and medicine.

Finally, through a review of current methods for evaluating intervention outcomes in selected studies, we identified a lack of evidence on degrees, forms, and strategies of interaction suitable for the severity of pain and anxiety in medical treatments. Although VR is widely accepted as a viable distraction from pain, our review identified 3 studies (published between 2017 and 2018), where such distraction did not generate any significant difference in pain or anxiety. A closer look reveals that 2 of those studies relied on passive user engagement through videos to reduce pain and anxiety. We note that children are increasingly familiar with VR technology, and perhaps, a lack of technology novelty and interactive feedback is responsible for those failed interventions. A link between age and response to VR interactivity should be considered, as research has shown a significant increase in pain tolerance in children aged 10 to 15 years who used VR compared with younger children aged 6 to 9 years [20].

### Limitations

A notable limitation in reviewed articles is a lack of assessment measures for establishing the efficacy of novel VR interventions for children and adolescents. Only 10 of 18 studies conducted testing or trials on the efficacy of VR apps. These studies consistently used self-report to establish the success of the intervention based on anxiety, pain severity, stress, or worry associated with pain variables. Only 1 study [24] addressed pain as a multifaceted experience with cognitive, affective, and perceptive components. This highlights a research gap on the impact of design elements (product or experience aspects) and strategy (distraction, focus shifting or skill building) on the pain or anxiety outcome, challenging the reliability of outcomes. Future research should use product, experience, and intervention aspects to describe the design and assess the outcomes in controlled trials and combine self-report with physiological indicators of pain and anxiety to determine efficacy. In addition, it is important to investigate differences between VR intervention goals (eg, distraction vs skill building) in relation to treatment outcomes. Overall, our observation leads us to believe there is a lack of interdisciplinary collaboration and research in relation to medical VR technology for pain and anxiety management, as a number of publications that involved a bespoke VR app did not present clinical testing, whereas the clinical trials consistently used commercial products and did not observe the impact of design on the intervention outcome.

### Conclusions

VR has been used widely in the health sector and has been demonstrated to be effective for modulating pain and anxiety in a range of settings, including pediatrics. A scoping review of 18 papers published between 2013 and 2018 revealed that VR has been adopted as a viable intervention for managing pediatric pain and anxiety in a range of medical treatments. However, only half of the reviewed studies attempted to design the VR app specifically for the context it was intended for. Consequently, there are limited guidelines to inform the design of effective VR apps for supporting patients in the pediatric setting. We propose that a holistic approach to designing VR in health care involves consideration of product, experience, and intervention aspects. We also propose future VR interventions to explore skill-building goals in apps characterized by dynamic feedback to the patient and experiential and product qualities that enable them to be an active participant in managing their own care.

## Appendix

Multimedia Appendix 1 Search strategies used on databases Scopus, ACM and PsycINFO.

Multimedia Appendix 2 A summary of reviewed articles.

## Abbreviations

ACM: Association for Computing Machinery
HMD: head-mounted display
MRI: magnetic resonance imaging
RQ: research question
VE: virtual environment
VR: virtual reality
