# Supplementary material for: Design Strategies for Virtual Reality Interventions for Managing Pain and Anxiety in Children and Adolescents: Scoping Review
Source: JMIR Serious Games. 2020 Jan 31;8(1):e14565. doi: 10.2196/14565 (PMC7055787; doi:10.2196/14565)
Supplement: Multimedia Appendix 1 [file games_v8i1e14565_app1.docx]

**Appendix 1 – search strategies**

The search term used for ACM are shown below, which yielded 1,010 unique articles:

( TITLE-ABS-KEY ( child* OR paediatric* OR pediatric* OR adolescen* ) AND PUBYEAR > 2012 ) AND ( TITLE-ABS-KEY ( "VIRTUAL REALITY" AND vr ) AND PUBYEAR > 2012 )

The search term used for Scopus is shown below, which yielded 315 articles:

( TITLE-ABS-KEY ( child* OR paediatric* OR pediatric* OR adolescen* ) AND PUBYEAR > 2012 ) AND ( TITLE-ABS-KEY ( "VIRTUAL REALITY" AND vr ) AND PUBYEAR > 2012 )

A detailed search strategy was developed for PsycINFO as shown in Table 1.

Table 1 Electronic database search for PsychINFO

| **Search ID number** | **Search terms** | **Results** |
| --- | --- | --- |
| 1 | \|  \|  \| \| --- \| --- \|   (paediat* or child* or adolescen*).mp. [mp=title, abstract, heading word, table of contents, key concepts, original title, tests & measures] | 838,531 |
| 2 | Limit 1 to (English language and yr=”2013-current”) | 183,685 |
| 3 | ("virtual reality" and VR).mp. [mp=title, abstract, heading word, table of contents, key concepts, original title, tests & measures] | \|  \| \| --- \|   1,570 |
| 4 | \|  \|  \|  \| \| --- \| --- \| --- \|   limit 3 to (english language and yr="2013 -Current") | 649 |
| 5 | 2 and 4 | 61 |
| 6 | from 5 keep 1-61 | 61 |
