# Supplementary material for: Design Strategies for Virtual Reality Interventions for Managing Pain and Anxiety in Children and Adolescents: Scoping Review
Source: JMIR Serious Games. 2020 Jan 31;8(1):e14565. doi: 10.2196/14565 (PMC7055787; doi:10.2196/14565)
Supplement: Multimedia Appendix 2 [file games_v8i1e14565_app2.docx]

**Multimedia Appendix 2.** A summary of reviewed articles.

| Author (year) | Medical procedure | N | Description of virtual reality app | Dependent variable | Assessment tools | Efficacy and testing result |
| --- | --- | --- | --- | --- | --- | --- |
| Grishchenko et al [34] | Medical procedures, broadly defined | Not applicable | Child travels on boat and exhales to move the boat. Some game modules need clinician prompting. | Pain and anxiety | —^a^ | — |
| Liszio and Masuch [17] | MRI^b^ examinations | 20 children and 4 staff | A penguin is exploring the MRI machine. The child plays games to learn about the MRI procedure as well. | Anxiety and UX^c^ | State-Trait Anxiety Inventory for Children and a nonstandardized UX questionnaire | No significant difference between control and experiment groups on trait anxiety and high perception of fun. |
| Faber et al [18] | Wound debridements | 36 | SnowWorld (commercial) | Worst pain intensity | Visual analog thermometer | Significantly less pain between baseline and days 1 to 3. No significant difference on subsequent days. |
| Hoffman et al [19] | Wound debridements—head wound | 1 | SnowWorld (commercial) | Pain unpleasantness, worst pain intensity, and fun | Global Rating Scale ratings | Less pain unpleasantness and intensity during VR^d^, more fun during physical therapy in VR than no VR conditions. |
| Jeffs et al [20] | Burn wound care | 28 | SnowWorld (commercial) | Pain, state anxiety, trait anxiety, and UX | Adolescent Pediatric Pain Tool and Spielberger State-Trait Anxiety Inventory for Children | Significantly less pain in VR group than a movie-viewing group, less pain than control but not significantly so |
| Piskorz and Czub [23] | Venipuncture | 38 | Multiple object tracking: child tracks flashing objects, controls the game using head movements. | Pain and stress | Self-created visual analogue scale (pain and stress rated on a scale of 0-10) | Significantly less pain and stress reported by intervention than control group. |
| Sil et al [27] | Cold pressor experiment | 62 | Nintendo Wii *Sonic and the Secret Rings* game (commercial) | Monitoring and blunting coping styles and pain tolerance | Game was played with and without VR in 2 cold pressor trials. Measured hand, Children’s Behavioral Style Scale, and pain tolerance recorded in duration (seconds) participants kept their hand in the cold water | Significantly better pain tolerance during both interactive videogame distraction conditions. There was no difference in differential response to videogame distraction with or without the enhancement of VR technology. |
| O’Sullivan et al [28] | Anesthesia in hospitals | — | How to film a 360° video of operation room | — | — | — |
| Birnie et al [30] | Implantable venous access device | 17 (in 3 phases) | Simulated underwater game of shooting rainbow balls at creatures and searching for treasures | Pain and anxiety | Pain (11-point numeric scale), stress (11-point numeric scale), and nausea (4-point scale) | Game perceived easy to use by most, who felt relaxed. All but 1 participant would use it again. No adverse effect reported. |
| Gold and Mahrer [25] | Blood draw | 143 triads | Bear Blast (commercial)—adventure and using gaze to fire a cannon at objects | Pain, anxiety, and satisfaction | Visual Analogue Scale for pain and anxiety, Colored Analogue Scale for pain, and Faces Pain Scale-Revised (worry related to pain). Facial Affective Scale, anxiety sensitivity, presence scales, Malaise Scale, and self-developed satisfaction scales | Pre- and postassessment: VR significantly reduced pain and anxiety. Significantly better affect in VR condition. High levels of immersion and satisfaction in VR, no adverse effect. |
| Atzori et al [24] | Venipuncture | 23 | SnowWorld (commercial) | Pain, quality of experience, and fun | Rating scales on cognitive time spent thinking about pain, affective unpleasantness, worst pain, and presence and realism of VR objects | Significantly lower pain unpleasantness and less time spent thinking about pain in VR condition, a strong sense of presence, significantly higher levels of fun, no significant differences in *worst pain* between subjects. |
| Chau et al [32] | Botulinum toxin for cerebral palsy | 14 | Commercial 360° videos on YouTube | Pain | FLACC^e^, scale range 0 to 2 in each category, feedback from caregiver | 9 caregivers wanted to use VR again, with patient scores 1-8 (median 2.5), and 2 caregivers were neutral. Overall subjective feedback was positive. |
| Ng et al [31] | Pediatric chemotherapy | 6 | A virtual farm, player cares for vegetables and animals | UX | Subjective feedback in focus groups | Participants enjoyed the form. Usability concerns included mobility with an IV, accuracy of gestures with cold hands, and need for more challenging game play. |
| Gerçeker et al [26] | Phlebotomy | 121 | Commercial 360° videos | Pain | Wong-Baker Faces pain rating scale (for reports by the child, parent, nurse, and the researcher) | No statistical difference between VR and another intervention (external cold and vibration). There was a significant difference for both intervention groups, compared with the control group. |
| Al-Halabi et al [33] | Dental procedure: inferior alveolar nerve block | 101 | Commercial 360° videos | Pain | Pain assessed based on self-report, pulse rate, and behavior. Wong-Baker Faces pain rating scale, FLACC behavior rating scale through external evaluator | No significant differences among groups on pain (Faces) and behavior (FLACC), a significant difference in pulse rate between VR and control group. VR intervention was more acceptable in older patients. |
| Scapin et al [21] | Burn dressing | 2 | Commercial videos (a roller coaster ride and a tour of a marine environment) | Pain | Facial and numeric scales (0-10), pain intensity, semistructured interviews, and observation | VR was effective in reducing the pain, VR was accepted, and easy to use. |
| Ko et al [22] | Wound dressing and motor rehabilitation in burn patients | — | A VR train ride of an arctic environment, with animal characters to guide rehabilitation | — | — | — |
| Ryu et al [29] | Perioperative care before anesthesia in hospital | 69 | A 360° video of person dressed as Pororo (a penguin) giving a tour of the operating theater | Anxiety, induction compliance, and procedural behavior | Modified Yale Preoperative Anxiety Scale, ICC^f^, and procedural behavior scale (PBRS^g^) | Significantly lower anxiety and lower ICC and PBRS in VR group than control, exhibiting efficacy in reducing anxiety and improving compliance |

^a^—: Not applicable

^b^MRI: magnetic resonance imaging.

^c^UX: user experience.

^d^VR: virtual reality.

^e^FLACC: Face, Leg, Activity, Cry and Consolability scale.

^f^ICC: Induction Compliance Checklist.

^g^PBRS: Pediatric Behavior Rating Scale.
